# Dexamethasone induces apoptosis in pulmonary arterial smooth muscle cells

**DOI:** 10.1186/s12931-015-0262-y

**Published:** 2015-09-18

**Authors:** Laura C. Price, Dongmin Shao, Chao Meng, Frederic Perros, Benjamin E. Garfield, Jie Zhu, David Montani, Peter Dorfmuller, Marc Humbert, Ian M. Adcock, Stephen J. Wort

**Affiliations:** Pulmonary Hypertension Service, Royal Brompton Hospital, London, SW3 6NP UK; Service de Pneumologie et Réanimation Respiratoire, Hôpital Bicêtre, 78 rue du Général Leclerc, 94270 Le Kremlin-Bicêtre, France; Department of Histopathology, Royal Brompton Hospital, London, SW3 6NP UK; Airways Disease Section, National Heart and Lung Institute, Imperial College London, Dovehouse Street, London, SW3 6LY UK; Department of Geriatrics, Renji Hospital, School of Medicine, Shanghai Jiaotong University, Shanghai, China

## Abstract

**Background:**

Dexamethasone suppressed inflammation and haemodynamic changes in an animal model of pulmonary arterial hypertension (PAH). A major target for dexamethasone actions is NF-κB, which is activated in pulmonary vascular cells and perivascular inflammatory cells in PAH. Reverse remodelling is an important concept in PAH disease therapy, and further to its anti-proliferative effects, we sought to explore whether dexamethasone augments pulmonary arterial smooth muscle cell (PASMC) apoptosis.

**Methods:**

Analysis of apoptosis markers (caspase 3, in-situ DNA fragmentation) and NF-κB (p65 and phospho-IKK-α/β) activation was performed on lung tissue from rats with monocrotaline (MCT)-induced pulmonary hypertension (PH), before and after day 14–28 treatment with dexamethasone (5 mg/kg/day). PASMC were cultured from this rat PH model and from normal human lung following lung cancer surgery. Following stimulation with TNF-α (10 ng/ml), the effects of dexamethasone (10^−8^–10^−6^ M) and IKK2 (NF-κB) inhibition (AS602868, 0–3 μM (0-3×10^−6^ M) on IL-6 and CXCL8 release and apoptosis was determined by ELISA and by Hoechst staining. NF-κB activation was measured by TransAm assay.

**Results:**

Dexamethasone treatment of rats with MCT-induced PH *in vivo* led to PASMC apoptosis as displayed by increased caspase 3 expression and DNA fragmentation. A similar effect was seen i*n vitro* using TNF-α-simulated human and rat PASMC following both dexamethasone and IKK2 inhibition. Increased apoptosis was associated with a reduction in NF-κB activation and in IL-6 and CXCL8 release from PASMC.

**Conclusions:**

Dexamethasone exerted reverse-remodelling effects by augmenting apoptosis and reversing inflammation in PASMC possibly via inhibition of NF-κB. Future PAH therapies may involve targeting these important inflammatory pathways.

## Introduction

Pulmonary arterial hypertension (PAH) is an incurable condition associated with remodelling of resistance, pre-capillary pulmonary arterioles, subsequent right ventricular failure and premature death. Despite recent advances in the understanding of underlying genetic susceptibility of PAH, the exact underlying pathogenesis is unknown and the condition remains incurable. Recent evidence suggests that inflammation plays an important role in the pathogenesis of both animal models of PH and human PAH (including idiopathic PAH) [1–7]. As such, concentrations of circulating cytokines, such as IL-6, are raised in patients with idiopathic PAH and are of prognostic importance [8, 9]. Furthermore, perivascular inflammatory cells are observed in post-mortem and post-transplant histological specimens [10–12] and there appears to be dysregulation of circulating inflammatory cells [13]. In support of continuing inflammation being important we have recently demonstrated up-regulation of NF-κB signalling in endothelial cells, smooth muscle cells, macrophages and lymphocytes in histological sections from patients with idiopathic PAH [5].

However, convincing evidence for anti-inflammatory or immunosuppressive therapy working in patients with PAH exists only in a minority: patients with mixed connective tissue disease, systemic lupus erythematosus, Castleman’s disease and Polyneuropathy, Organomegaly, Endocrinopathy, Monoclonal gammopathy and Skin abnormalities (POEMS) Syndrome [14–17]. Immunosuppressive therapy does not appear to be effective in scleroderma PAH [16]. To our knowledge immunosuppressive therapy has not been formally tested in patients with idiopathic PAH, although cases have been reported [18].

We have recently demonstrated that the glucocorticoid (GC) dexamethasone was able to prevent and reverse pulmonary vascular remodelling associated with the monocrotaline (MCT) model of pulmonary hypertension [19]. Dexamethasone also prevented and reversed the severe pulmonary haemodynamics associated with this model of pulmonary hypertension [19]. Furthermore, we were able to show that dexamethasone inhibited proliferation of pulmonary arterial smooth muscle cells (PASMC) isolated from this model [19]. In an isolated report, prednisolone appeared to inhibit proliferation of PASMC from patients with idiopathic PAH, associated with a reduction in cell cycle markers [20]. However, inhibition of proliferation does not explain the reversal of remodelling we observed in the MCT model of PH and would not provide the optimal potential therapy for patients who are likely to have significant remodelling of their pulmonary vasculature at diagnosis. As such we sought to investigate the mechanisms by which GCs reverse remodelling in the MCT model of PH. Understanding such mechanisms may provide novel and more effective treatments for the future.

## Materials and methods

### In situ DNA fragmentation assay

In situ DNA fragmentation was performed on paraffin lung sections using a VasoTACS kit (R&D systems) according to Manufacturer’s instructions. The TACS-XL assay uses Terminal deoxynucleotidyl Transferase (TdT) to incorporate nucleotides into the 3′-OH ends of DNA fragments. These nucleotides are BrdU-labeled and a biotinylated anti-BrdU antibody is then used for detection.

### Rat lung immunohistochemistry

Rat lung Paraffin sections (5 μm thick) were obtained following *in vivo* experiments as previously described [19]. Sections were incubated with peroxidase blocking solution (Dako, Cambridge) and then with primary antibodies for rabbit anti-active caspase-3 (1:50 Abcam ab2302), NF-κB p65 (1:200 Cell Signaling C22B4), rabbit anti-phospho-IKKα/β (1:40 Cell Signaling 2697), P-Stat3 (1:50 Cell Signalling 9145); Stat3 (1:400 Cell Signalling 9149) or SMA (1:400 Dako M0851). Sections were then incubated with polyclonal goat anti-rabbit horseradish peroxidase (HRP)-conjugated secondary antibody (Dako) followed by incubation with diaminobenzidine (DAB) and peroxide buffer (Sigma) to produce a brown stain. Slides were counterstained with hematoxylin or eosin to provide nuclear and morphological detail. Non- specific rabbit IgG (Sigma-Aldrich) at the same concentration as those used above was used as a control.

### Immunohistochemical scoring

Slides were numbered and coded and performed by two blinded assessors. Cells within pulmonary vessels were identified using light microscopy. Standard morphometric end points were also measured. At least 10 sections per subject were analyzed. For apoptosis, the percentage (e.g. for caspase-3) of pulmonary arterial smooth muscle cells (PASMC) staining positive was determined over the total number of cells counted.

### Pulmonary arterial smooth muscle cell isolation and culture

PASMCs were isolated from control and monocrotaline (MCT)-exposed rats and from normal human lung excised from patients undergoing surgery for lung cancer as previously described [19, 21]. For the MCT model, male 6-week old Wistar rats (100 g body weight) were maintained in a temperature-controlled room with a 12:12-h light–dark cycle with access to standard rat chow and water. Rats received a standard single subcutaneous injection of 60 mg/kg monocrotaline (MCT) (Sigma-Aldrich, Lyon, France) [19]. Rat PASMCs were maintained in DMEM containing 10 % FCS and used from passage 3–6. Human PASMCs were maintained in DMEM containing 15 % FCS and used from passage 3–8.

### Hoechst 33342 staining

Rat and human PASMCs were plated at a seeding density of 2.5×10^4^ cells/well in 8-well Lab-Tek slides. Cells were starved for 24 h and then treated with various concentrations of dexamethasone or IKK-2 inhibitor (AS602868, kindly provided by Serono International SA, Geneva, Switzerland) for 24, 48 and 72 h in DMEM without FCS. After treatment, cells were fixed and mounted with solution containing 5 μg/ml Hoechst 33342. Images (3–5 non-overlapping randomly selected fields for each well) were taken using a Zeiss fluorescence Microscope. The number of apoptotic cells exhibiting condensed nuclear fluorescence was determined and expressed as a proportion of the total cells.

#### ELISA for DNA fragmentation

The Cell Death Detection ELISA Plus kit (Roche Applied Science, Indianapolis, USA) was used to detect cytoplasmic histone-associated-DNA fragments. PASMC were plated at 5×10^4^ cells per well in 24-well plates and treated as described above. After treatment, cells were lysed with lysis buffer. ELISA was performed according to manufacturer’s instruction. The samples are placed into a streptavidin-coated microplate and incubated with a mixture of anti-histone-biotin and anti-DNA-peroxidase. Nucleosomes are captured via their histone component whilst anti-DNA-peroxidase binds to the DNA part of the nucleosomes. After removal of the unbound antibodies, the amount of peroxidase retained in the immunocomplex is photometrically determined with ABTS as the substrate. This allows the specific determination of mono- and oligonucleosomes in the cytoplasmic fraction of cell lysates.

### Measurement of cytokine release from human PASMC

Human PASMC were plated in 96-well plates (5000 cells/well) and cultured in DMEM/15 % FCS until 90 % confluent; cells were serum-starved overnight and then tumour necrosis factor (TNF)-α (10 ng/ml) for 24 h used as the stimulus in the presence or absence of dexamethasone (10^−8^–10^−6^ M) or an IKK-2 inhibitor (AS602868, 0–3 μM). After 24 h, the supernatant was aspirated and stored at −20 °C and MTT viability assays (Sigma Aldrich) were performed. ELISA measurement of IL-6 and CXCL8 levels (Human ELISA DuoSet, R&D Systems) were performed on supernatants according to the manufacturer’s instructions as follows using distinct capture and detection antibodies: 100 μl of assay diluent, 50 μl of standard or cell culture supernatants were pipetted to each well sequentially, and incubated at room temperature for 2 h. After incubation, wells were washed 4 times and incubated with 100 μl of conjugate for 1 h. Wells were then washed 4 times and incubated with 200 μl of substrate solution for 30 min. The plates were read at 450 nm using a BioTek plate reader immediately after 50 μl of stop solution was pipetted to each well.

### Western blot analysis

150 mg of rat lung tissue from each animal was cut into 1 mm^3^ size pierces and put into a CK14 Precellys Lysing kit (Stretton Scientific), 150 μl 2× cell lysis buffer (Cell signaling) was then added to each tube, tissue lysis was performed using Precellys 24 (Stretton Scientific) program 1 (5100 rpm 2×15s pause 20 s). Samples were then centrifuged at 13000 rpm for 1 min; supernatants were centrifuged again at 13000 rpm for 10 min. 40 μg of total protein from each sample was separated on SDS-PAGE and transferred to nitrocellulose membranes or polyvinylidene fluoride membranes (GE Healthcare). Membranes were probed with anti- activated caspase 3, and GAPDH (Cell Signaling). Membranes were developed using ECL (GE Healthcare). The intensity of the bands was quantified using Image J (NIH).

### Measurement of activation of NF-κB

#### Tissue preparation and nuclear extraction

Human PASMC (1x10^6^ cells) were plated in 10 cm cell culture dishes overnight and then starved for 24 h. After starvation, cells were treated with TNF-α (0–10 ng/ml) in the presence or absence of dexamethasone (10^−7^ M) for 24 h. Cells were then collected and resuspended in 100 μl of 1X hypotonic buffer and incubated on ice for 15 min. After incubation, 5 μl detergent was pipetted to cell suspension and vortex 10 s at the highest setting. Cell suspensions were then centrifuged at 14,000 × g for 30 s at 4 °C and supernatants were removed. The pellets (nuclears) were resuspended in 50 μl of complete lysis buffer and incubate on ice for 30 min. Nuclear suspensions were vortex for 30 s at the highest setting before centrifugation at 14,000 x g for 10 min (Active Motif, Rixensart, Belgium). The protein contents in the supernatants (nuclear extracts) were estimated using Bradford assay and then frozen at −80 °C.

#### NF-κB transcription factor p65 TransAM assay

This was performed on nuclear extracts using the p65 TransAM® Kit (Active Motif) according to the manufacturer’s instructions. The TransAM assay has an NF-κB consensus-binding site oligonucleotide immobilized to a 96 well plate. Nuclear extract is added to each well and activated NF-κB binds specifically to the bound oligonucleotide. This is detected using an anti-p65 subunit antibody specific for an epitope on the bound and active form of p65. Subsequent incubation with a secondary antibody and developing agent enables quantification.

#### Statistical methods

Data are presented as mean and standard error of the mean (SEM). For each dataset the normality of distribution was determined using the Kolmogorov-Smirnov test. Single comparisons were made using Students *t*-test, and multiple comparisons tested using one-way ANOVA, followed by appropriate post-tests. Differences at a *p* < 0.05 were considered significant. Statistical analysis used Prism version 5.0 (GraphPad software, La Jolla, CA, USA).

## Results

### Effects of dexamethasone on rat PASMC apoptosis: immunohistochemical analysis

**Fig. 1:**
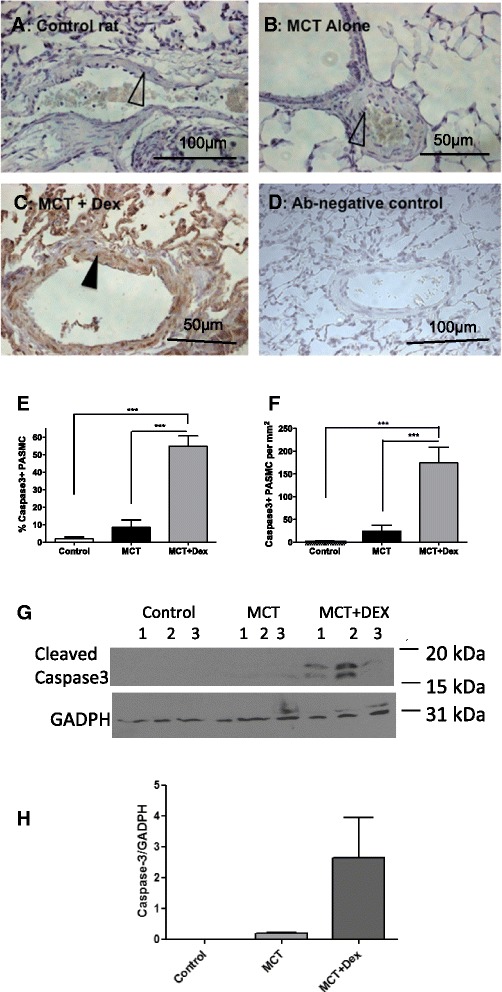
Dexamethasone increases caspase-3 immunohistochemistry scores in rat pulmonary artery smooth muscle cells (PASMC) *in vivo.* Caspase-3 immunostaining was performed on rat lung resected at day 28 in (**a**) control rats showing caspase-negative PASMC (unfilled arrow) (magnification ×100), (**b**) monocrotaline (MCT)-treated rats showing caspase-negative PASMC (unfilled arrow) (**b**) and (**c**) (B and C, magnification ×200), MCT+ dexamethasone (5 mg/kg/day, days 14–28)-treated rats showing caspase-positive PASMC (filled arrow). Images are representative of *n* = 5 per group. Antibody-negative staining controls are shown in an untreated control rat (**d**, magnification ×100). Caspase-3 immunohistochemistry scoring was performed by an assessor blinded to the groups and presented as (**e**) percentage of caspase-3 positive PASMC over total PASMC and (**f**) the numbers of caspase-3-positive PASMC per mm^2^. Western blot analysis of cleaved caspase 3 in 3 independent lung samples from control untreated, MCT-treated and MCT + dexamethasone (MCT + Dex)-treated rats (**g**). Densitometric analysis of the data is shown in (**h**) with GAPDH used as a loading control. Results represent mean ± SEM of 5 rats per group compared using Student *t* test. *p < 0.05, **p < 0.01, ***p < 0.001

Immunohistochemical analysis of rat lung demonstrated an increase in caspase-3 immunostaining (solid arrow) within the PASMC layer in dexamethasone-treated MCT rats at day 28 compared to untreated MCT controls (caspase-3-negative PASMC, unfilled arrows, Fig. 1a–c). When assessed by morphometric scoring, there was a significant increase in caspase-3-positive PASMC staining in the dexamethasone-treated MCT group compared to the untreated MCT group (8.4 ± 4.3 vs. 54.8 ± 5.9 %, *p* < 0.0001) (Fig. 1e–f). This observation was further confirmed by Western blot analysis for caspase 3 in whole lung protein extracts followed by densitometry analysis (Fig. 1g–h).

**Fig. 2:**
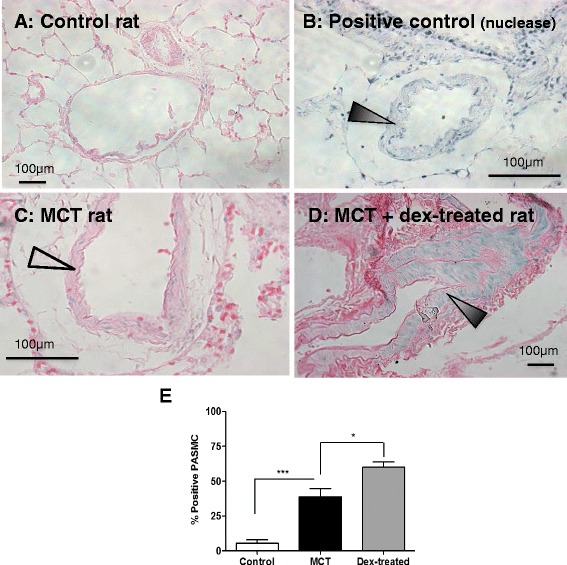
Dexamethasone increases apoptosis in pulmonary artery smooth muscle cell (PASMC) layer in monocrotaline (MCT) and dexamethasone-treated rats. In-situ detection of DNA fragmentation was performed on lung sections from control rats and on lungs resected from rats 28 days following MCT, with and without dexamethasone treatment (5 mg/kg/day from day 14–28). Representative examples in (**a**) a control rat, (**b**) nuclease-positive cells in an MCT-treated rat (black arrow), (**c**) negative-stained cells (unfilled arrow) in a rat at day 28 following MCT and (**d**) in a rat at day 28 following MCT and dexamethasone treatment (showing positive stained cells, black arrow). Results are representative of *n* = 5 per group (for **a**, **c** and **d**). Slides are counterstained with eosin. Magnification ×200. Scoring was performed by an assessor blinded to the groups for percentage positive PASMC over total number of counted cells (**e**). Results represent mean ± SEM of *n* = 5 per group, compared using 1-way ANOVA and Bonferroni’s post-test., *p < 0.05 ***p < 0.001

Further, *in-situ* DNA fragmentation assay showed that the percentage of PASMC scoring positive (an increase blue nuclear staining) increased. Blinded morphometric scoring in lung sections from rats showed that apoptosis was increased in MCT-treated controls compared to untreated rats (5.3 ± 2.8 vs. 38.8 ± 6.1, *p* < 0.001) (Fig. 2a, c, e) and was further increased in dexamethasone-treated MCT-exposed rats (38.8 ± 6.1 vs. 59.9 ± 3.9, *p* < 0.05) (Fig. 2c, d, e).

### Effects of dexamethasone on rat PASMC apoptosis: *in vitro* analysis

**Fig. 3:**
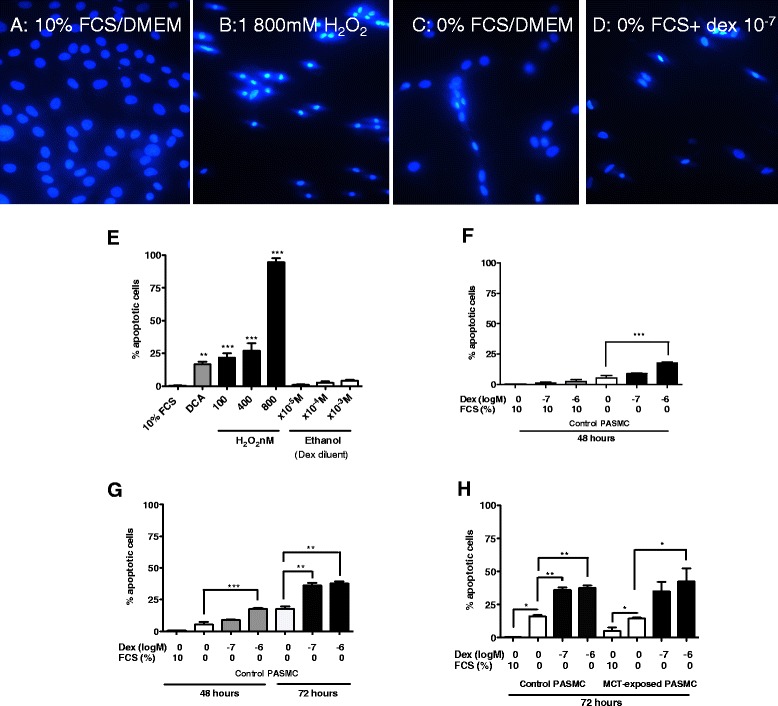
Effects of foetal calf serum (FCS) and dexamethasone on apoptosis of cultured control rat PASMC. Representative Hoechst 33342 stained images from PASMC from control rats treated for 48–72 h with (**a**) 10 % FCS/DMEM as a negative control, (**b**) 800nM hydrogen peroxide (H_2_O_2_) as a positive control, (**c**) 0 % FCS and (**d**) 0 % FCS plus dexamethasone (10^−7^ M). Images are representative of 5–8 images per condition from *n* = 3–4 independent experiments per group at a magnification ×400. Data from these and other similar experiments inducing apoptosis by treating cells with dichloroacetate (DCA, 100 μM) and differing concentrations of H_2_O_2_ (100-800nM) for 48 h are quantified in (**e**). The effect of altering FCS concentrations on dexamethasone-induced apoptosis of PASMC at 48 h are shown graphically in (**f**) in cells from control rats. (**g**) The increased apoptosis observed by culturing cells with FCS and dexamethasone for 72 h rather than 48 h. Similar apoptotic responses to dexamethasone and FCS are seen in PASMC isolated from control and 21 day MCT-exposed rats (**h**). Results represent mean ± SEM from *n* = 3–5 per group, compared using 1-way ANOVA and Bonferroni’s post-test. *p < 0.05, **p < 0.01, ***p < 0.001

Serum withdrawal (0 % FCS) induced a characteristic apoptotic response in PASMC, as demonstrated by nuclear condensation with Hoechst 33342 staining (Fig. 3). Dexamethasone (10^−7^ M and 10^−6^ M) increased this 0 % FCS-induced PASMC apoptosis at 48 and 72 h in a time- and concentration-dependent manner (Fig. 3f and g). There was no significant difference in the ability of serum starvation alone and/or dexamethasone treatment to increase apoptosis in cells from control or day 21-MCT-exposed rats at 72 h (Fig. 3h).

**Fig. 4:**
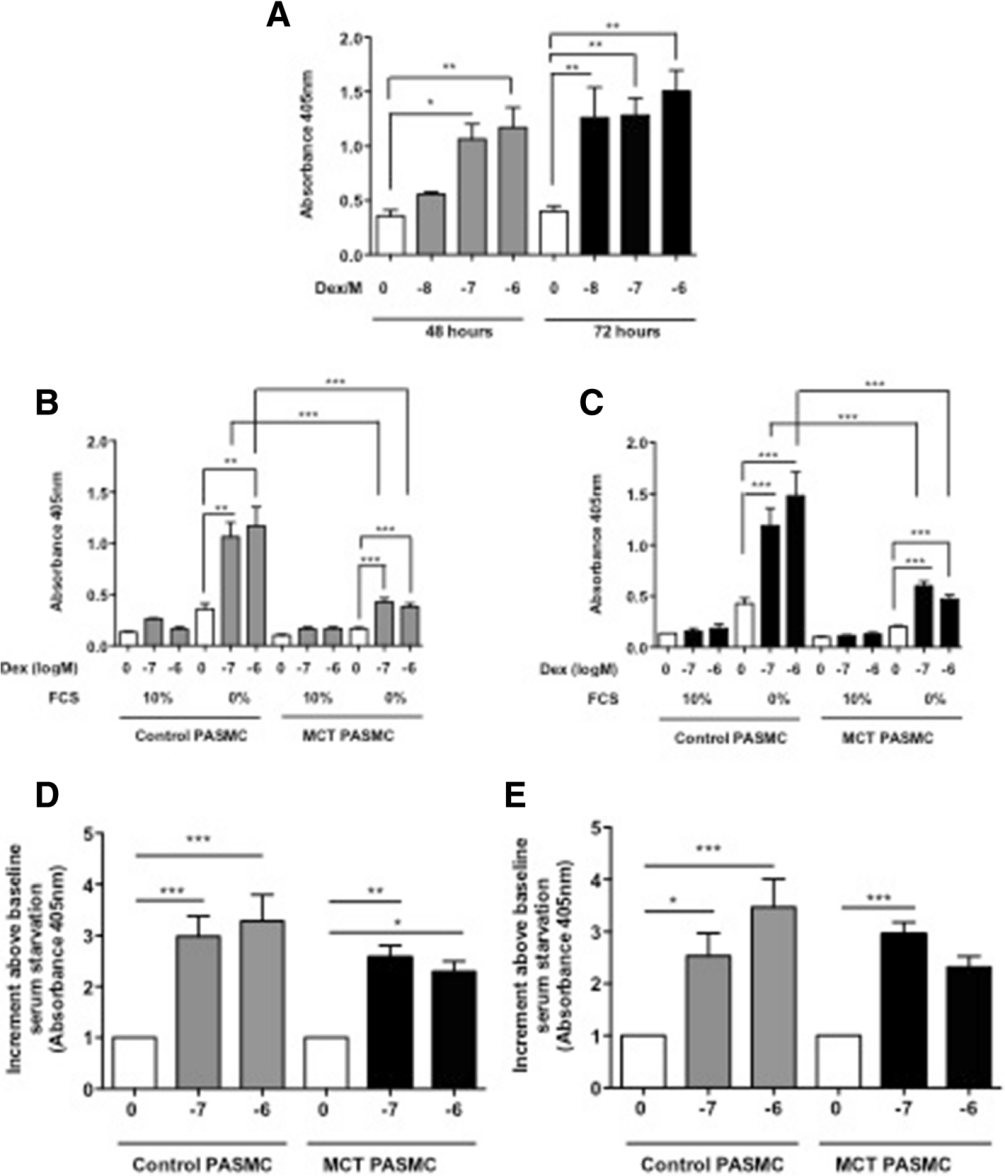
Dexamethasone increases apoptosis in 21 day monocrotaline (MCT)-treated rat pulmonary arterial smooth muscle cells (PASMC). Rat PASMC were isolated from control rats and from rats 21 days after exposure to MCT and treated with an increasing concentration of dexamethasone (Dex) and appropriate controls. Apoptosis, as measured by DNA fragmentation, was increased from baseline in serum-starved cells (0 % FCS) in control PASMC by Dex (10^−8^–10^−6^ M) at both 48 h (grey bars) and 72 h (black bars) (**a**). There was a significant difference between control and MCT-exposed cells at 48 h (**b**) and 72 h (**c**) following 10^−7^ M and 10^−6^ M Dex treatment. However this difference was lost when data were normalised to baseline serum starvation levels at 48 h (**d**) and 72 h (**e**). Results represent mean ± SEM from *n* = 3–5, compared using 1-way ANOVA and Bonferroni’s post-test. *p < 0.05, **p < 0.01, ***p < 0.001

To confirm these results, we analyzed DNA fragmentation using an ELISA-based assay. These results also showed that dexamethasone significantly increased apoptosis in a time- and concentration-dependent manner in serum-starved rat PASMC, especially in control cells, reaching a plateau after 72 h with 10^−8^ M dexamethasone (*p* < 0.01) (Fig. 4a). Dexamethasone increased apoptosis in PASMC from control rats and from rats exposed to MCT for 21 days at both 48 and 72 h. There was significantly less apoptosis induced in cells from MCT-treated rats compared to control rats in terms of absolute values (Fig. 4b and c). However, when expressed as the proportion of apoptosis from baseline serum starvation the difference was no longer significant (Fig. 4d and e).

### Effect of dexamethasone on NF-κB and Stat3 activation in rat pulmonary vascular cells

**Fig. 5:**
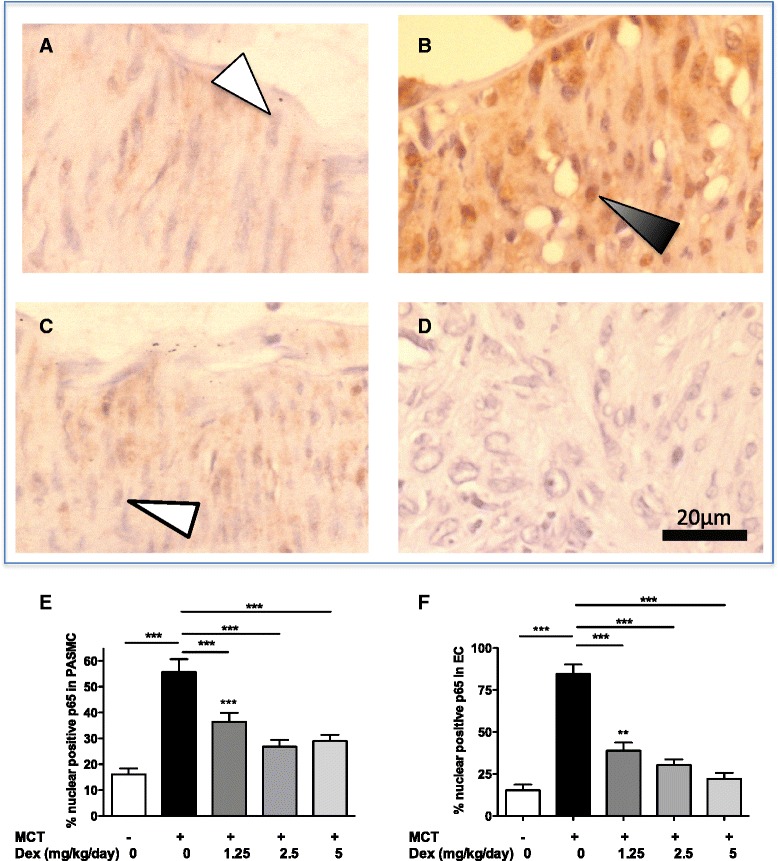
Dexamethasone reduced nuclear p65 expression in lung vascular cells of rats with monocrotaline (MCT)-induced pulmonary hypertension (PH). Rats with MCT-induced PH were treated with dexamethasone (Dex, 5 mg/kg/day) from day 14–28. Immunohistochemical staining for p65 (brown) was performed on lung sections from rats from untreated controls at day 28 (**a**) showing p65-ve cells (unfilled arrow). (**b**) p65 + ve cells (filled black arrow) were observed in MCT treated rats at day 28. In contrast, p65-ve cells (unfilled arrow) were seen in dexamethasone-treated MCT-exposed rats (**c**). An antibody-negative control is shown in slide **d**. Magnification × 200. Blinded morphometric scoring showed that MCT increased the percentage of total nuclear p65-positive cells in PASMC (**e**) and endothelial cells (**f**). This was reduced in all dexamethasone-treated groups in a dose-dependent manner. Results represent mean ± SEM of *n* = 5 per group, compared using Kruskal Wallis and Dunn’s post-test. *p < 0.01, **p < 0.01, ***p < 0.001

**Fig. 6:**
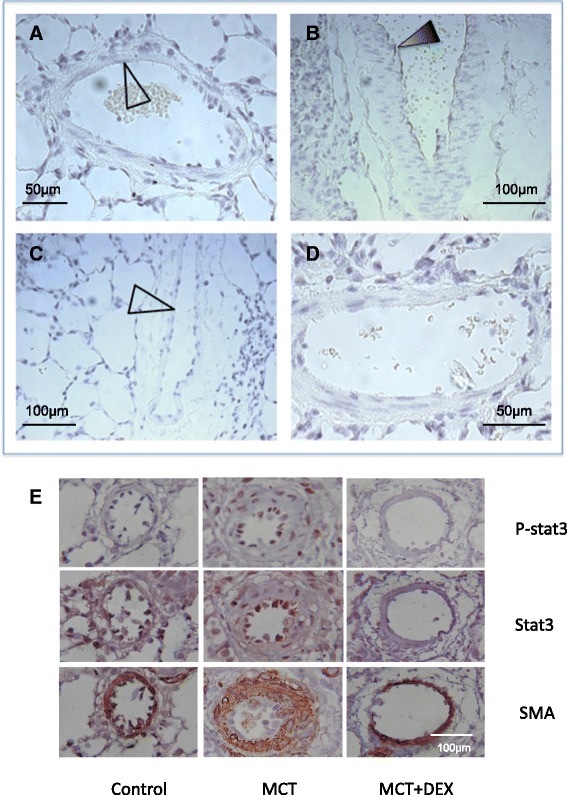
Dexamethasone reduces monocrotaline (MCT)-induced enhancement of phospho-IKK-α/β immunostaining in rat lung vascular cells. The expression of activated phospho-IKKα/β (brown staining) was measured in lung sections from rats from untreated control rats (**a**); day 28 MCT-treated rats (**b**) and MCT and dexamethasone day 14–28-treated rats (**c**). These data suggest an increase in IKKα/β activation in the EC layer in MCT rats (*black arrow*) (**b**), which was reduced in dexamethasone-treated rats (unfilled arrow) (**c**). Sections represent *n* = 2–3 per group (**a**–**c**); and were counterstained with haematoxylin. Antibody-negative control is shown in slide D. Magnification x100 (**b**, **c**) and ×200 (**a**, **d**). Phospho (P)-Stat3 and Stat3 staining was performed in control, MCT and MCT + Dex rats (*n* = 3 per group), with SMA staining of smooth muscle actin (bottom panel, magnification x200) (**e**). The results show that MCT enhances P-stat3 expression and that this is reduced in the presence of dexamethasone

We have previously shown activation of NF-κB in vascular cells and macrophages in end-stage human idiopathic PAH [5]. To determine whether elevation of NF-κB activity also plays a role in the development of rat MCT-PH, we performed morphometric analysis of NF-κB p65 in PASMC and endothelial cells (EC) in lung sections. As shown in Fig. 5, we observed an increase in nuclear p65 activation in lung from rats with MCT-induced PH at day 28 compared to control rats in both PASMC (16.1 ± 2.27 % vs. 55.7 ± 5.02 %, *p* < 0.001) and EC (15.3 ± 3.46 % vs. 84.4 ± 5.75 %, *p* < 0.001) (Fig. 5). Nuclear p65 activation was reduced at day 28 following day 14–28 dexamethasone treatment of the rats in both PASMC and EC (*p* < 0.001 for both cell types) at all doses of dexamethasone studied compared to MCT alone (Fig. 5). In addition, activation of the upstream activator of NF-κB, IKK-α/β, was increased in EC (although not PASMC) in the MCT-PH model and reduced by dexamethasone in lung tissue from the MCT-induced PH study (Fig. 6a–d).

Stat3 was also activated in the remodeled pulmonary artery (Fig. 6e). No activated phospho-Stat3 (P-Stat3) staining was observed in the lung from control rats whereas P-Stat3 was expression was clearly present in the lung of MCT-treated rats. Interestingly, total Stat3 staining was also increased in the lungs of MCT-treated rats. Dexamethasone treatment abolished the MCT-induced elevation of P-Stat3 expression as well as significantly reduced total Stat3 levels (Fig. 6e). Although, NFAT1 (NFATC2) has been reported to be elevated in pulmonary artery in the lung from iPAH patients [22], we were unable to detect NFAT1 in the lung from control and MCT rats (data not shown). It is possible that NFAT is not involved in this model or a different NFAT isoform is elevated in this model.

### The effect of dexamethasone and an IKK-2 inhibitor on IL-6 and CXCL-8 release from human pulmonary artery smooth muscle cells

**Fig. 7:**
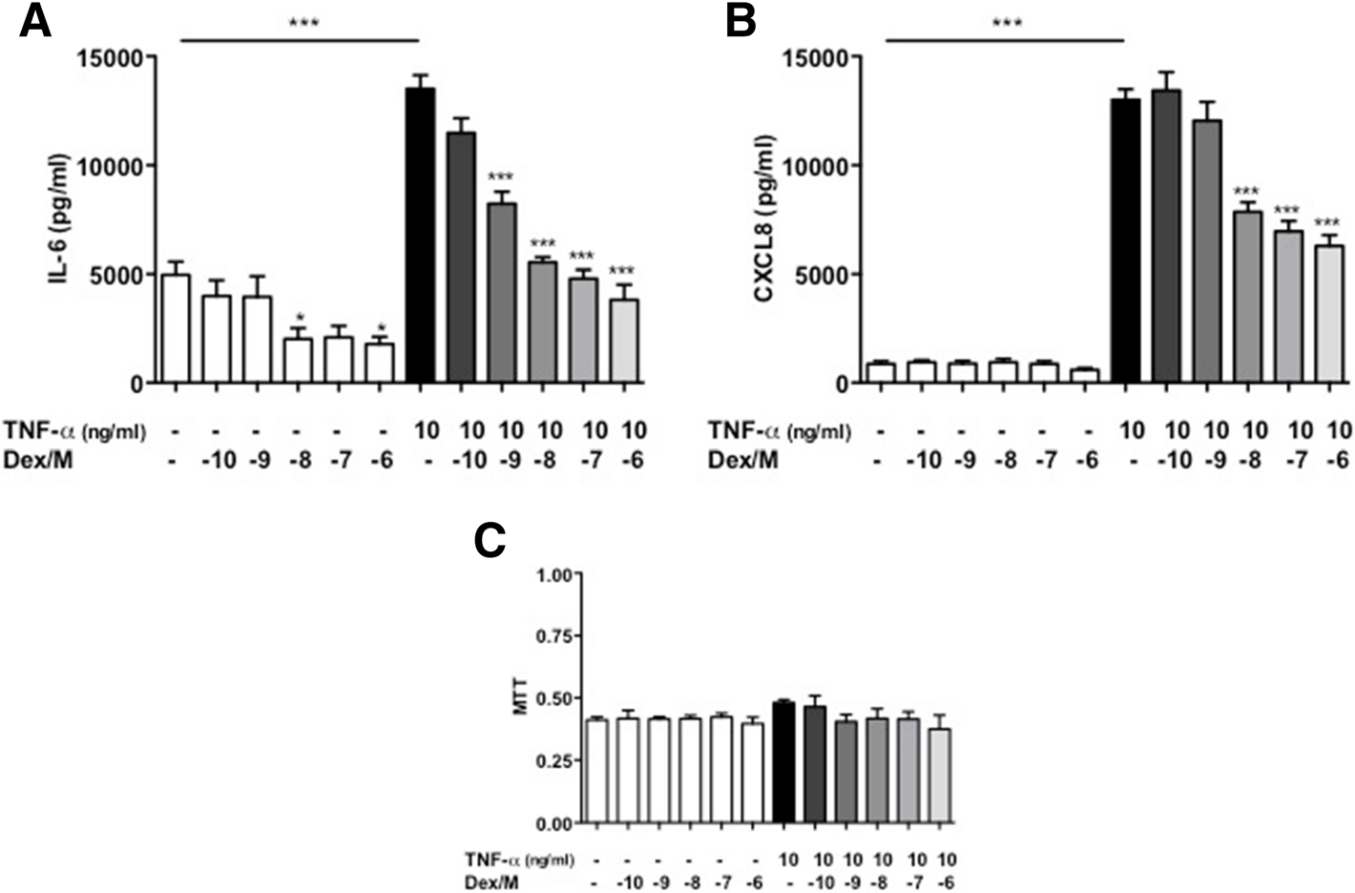
Dexamethasone (Dex) reduces interleukin (IL)-6 and CXCL8 release from human pulmonary arterial smooth muscle cells (PASMC). Human PASMC were plated out at 5000/well in 96-well plates; serum-starved overnight, and treated with tumour necrosis factor (TNF)-α (10 ng/ml) for 24 h in the presence and absence of dexamethasone (10^−10^–10^−6^ M) and IL-6 (**a**) and CXCL8 (**b**) release into the cell supernatant measured by ELISA. MTT assays were also performed to assess cell viability (**c**). Results represent mean ± SEM for *n* = 4, compared using 1-way ANOVA and Bonferroni’s post-test analysis. *p < 0.05, ***p < 0.001

We have previously shown in the MCT-induced PH model that dexamethasone treatment resulted in reversal of remodeling, and that this was associated with reduced IL-6 activity [19]. We were interested to determine the effect of dexamethasone and the IKK-2 inhibitor AS602868 on IL-6 production by human PASM cells in vitro. We selected to use TNF-α as our *in vitro* stimulus, as this is a well-known activator of NF-κB [23] as well as being implicated in PAH [24]. Dexamethasone and AS602868 both caused a concentration-dependent reduction in TNF-α-stimulated IL-6 and CXCL8 release from human PASMCs. IL-6 release was increased from baseline by TNF-α (4962 ± 581 vs 13056 ± 624 pg/ml, *p* < 0.001) and was reduced in a concentration-dependent manner by dexamethasone (10^−9^–10^−6^ M, *p* < 0.001) (Fig. 7a). CXCL-8 release was similarly increased by TNF-α (866 ± 127 vs 13018 ± 464 pg/ml, *p* < 0.001) and was reduced in a concentration-dependent manner by dexamethasone (10^−8^–10^−6^ M, *p* < 0.001) (Fig. 7b). Although IL-6 and CXCL8 are not strictly relevant to the study of apoptosis, they are critical to understanding the importance of inflammation in PAH and are also useful readouts of NF-κB activity.

**Fig. 8:**
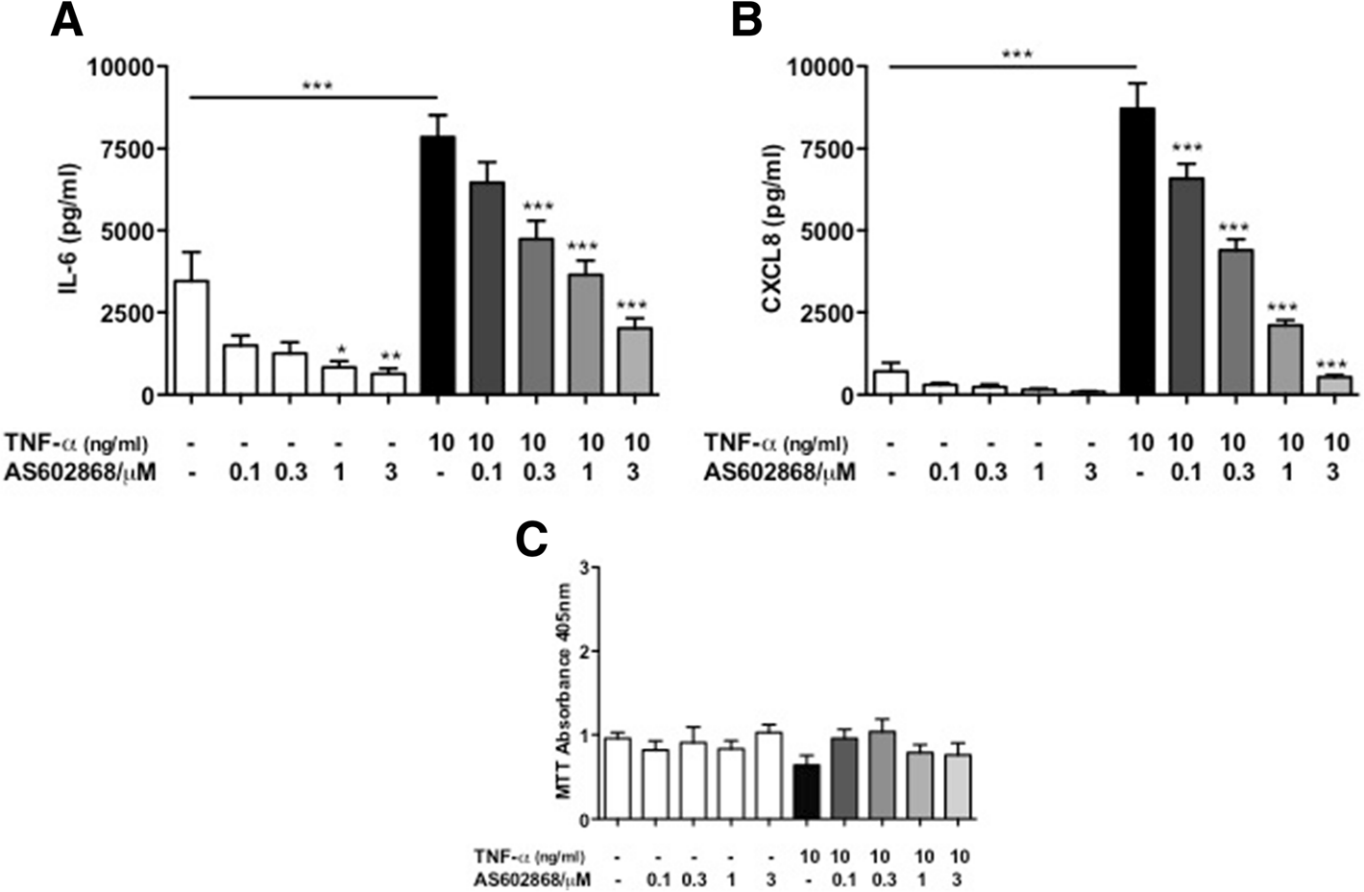
IKK-2 inhibition reduces interleukin (IL)-6 and CXCL8 release from human pulmonary arterial smooth muscle cells (PASMC). Human PASMC were plated at 5000/well in 96-well plates; starved overnight in FCS-free media and treated with tumour necrosis factor (TNF)-α (10 ng/ml) with and without the IKK-2 inhibitor AS602868 (0–3 μM) for 24 h. IL-6 (**a**) and CXCL8 (**b**) release into the supernatants was measured by ELISA. MTT assays were also performed to assess cell viability (**c**). Results represent mean ± SEM for *n* = 4, compared using 1-way ANOVA and Bonferroni’s post-test. *p < 0.05, ***p < 0.001

Similarly, the increase in IL-6 release by TNF-α (3450 ± 887 vs 7856 ± 657 pg/ml, *p* < 0.001) was also reduced in a concentration-dependent manner by AS602868 (0.3–3 μM, *p* < 0.001) (Fig. 8a). CXCL8 release was enhanced by TNF-α (702 ± 266 vs 8701 ± 775 pg/ml, *p* < 0.001) and again was reduced by AS602868 in a concentration-dependent manner (0.1–3 μM, *p* < 0.001) (Fig. 8b). For all these experiments, PASMC viability was assessed by MTT and showed no change in cell viability across all conditions (Figs. 7c and 8c).

### Dexamethasone inhibits NF-kB activity in human pulmonary artery smooth muscle cells

**Fig. 9:**
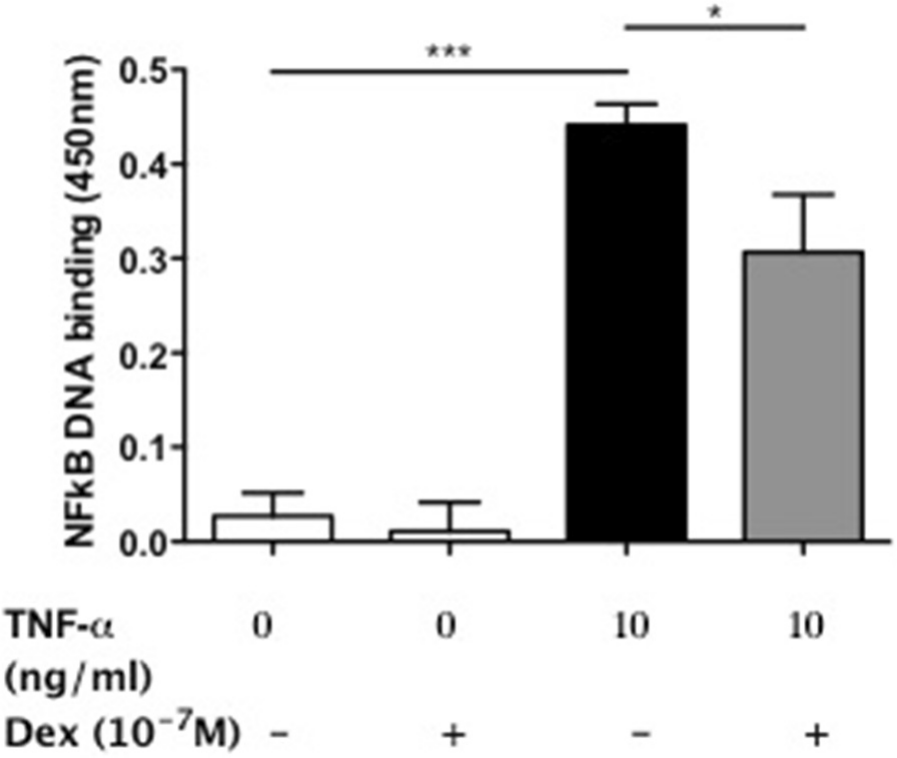
Dexamethasone (Dex) inhibits tumour necrosis factor (TNF)-α-induced activation of nuclear factor (NF)-κB in human pulmonary arterial smooth muscle cells (PASMC). PASMC from 3 healthy human subjects were stimulated with TNF-α (0–10 ng/ml) in the presence and absence of dexamethasone (Dex, 10^−7^ M) for 1 h. NF-κB p65 DNA binding in nuclear extracts was measured by TransAm assay. Data are reported as the mean ± SEM. *p < 0.05, ***p < 0.001 using Kruskal Wallis and Dunn’s post-test analysis

As one of the major mechanisms of action of dexamethasone is through inhibition of NF-κB activity, we performed a TransAM assay to measure NF-κB p65 DNA binding in nuclear extracts, using a selected single concentration of dexamethasone (10^−7^ M). Following TNF-α simulation there was an increase in NF-κB activity (0.027 ± 0.025 to 0.44 ± 0.022 units, *p* < 0.001), which was significantly reduced following treatment with 10^−7^ M dexamethasone (0.44 ± 0.022 to 0.31 ± 0.062 units, *p* < 0.01) (Fig. 9). This would indicate that at least part of the effects of dexamethasone in human PASMC is through inhibition of p65 NF-κB DNA binding but other aspects of the NF-κB pathway may also be affected.

### The effect of IKK-2 inhibition on apoptosis of human PASMCs

**Fig. 10:**
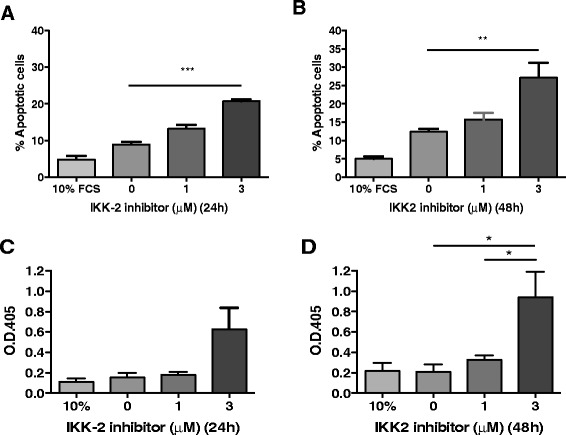
IKK2 inhibition increases apoptosis of human pulmonary arterial smooth muscle cells (PASMC). Human PASMC were isolated from normal human lung following surgery and treated with an increasing concentration of the IKK2 inhibitor AS602868 (0–3 μM). Apoptosis as determined by Hoechst staining was increased from baseline in serum-starved cells (0 % FCS) at 24 h (**a**) and 48 h (**b**) (*n* = 3 for each group). Similar results were seen when apoptosis was measured at 24 h (**c**) and 48 h (**d**) using a DNA fragmentation ELISA (*n* = 5 for each group). Results represent mean ± SEM, compared using 1-way ANOVA and Bonferroni’s post-test. *p < 0.05, **p < 0.01, ***p < 0.001

To directly link inhibition of NF-κB with the observed increase in apoptosis seen in rat PASMs *in vivo* and *in vitro*, we investigated the effect of IKK2 inhibition on apoptosis of human PASMC *in vitro*. After serum deprivation, Hoechst staining demonstrated increased human PASMC apoptosis following IKK2 inhibition (3 μM) from 9.23 (7.43–10.08) % in controls to 20.74 (19.8–21.6) % with 3 μM IKK2 inhibitor (*p* < 0.001) at 24 h, and from 13.42 (10.8–13.4) % to 26.9 (20.3–34.3) % at 48 h (*p* < 0.01) (Fig. 10a-b). A second apoptosis measurement technique using an ELISA-based assay to detect DNA fragmentation similarly demonstrated an increase in PASMC apoptosis following IKK2 inhibition (0.21 ± 0.07 vs. 0.94 ± 0.25, *p* < 0.01 at 48 h) (*n* = 5) (Fig. 10c-d).

## Discussion

In this study, using *in vivo* and *in vitro* models, we have shown that the reverse-remodelling effect of dexamethasone, previously observed in our MCT-treated rat model [19], involves, at least in part, increased apoptosis of PASMC. Furthermore, we show, for the first time, that dexamethasone treatment caused apoptosis of human PASMC *in vitro* as indicated by increased caspase 3 activation and DNA fragmentation.

Dexamethasone has been previously shown to induce apoptosis in several immune cells including T and B-lymphocytes, monocytes, eosinophils [25] and both activated and non-activated PBMC [26]. In these cells, dexamethasone-induced apoptosis is thought to play a homeostatic or immunosuppressive role [27]. Dexamethasone also induces apoptosis in non-immune cells including nasal polyp fibroblasts [28], but generally is protective against apoptosis in non-immune cells including mammary cells, human gastric cancer cells, lung epithelial cells and hepatoma cells and thereby protects them from ongoing inflammatory damage [25]. The pro- or anti-apoptotic effect of dexamethasone is thus considered cell type-dependent.

Proposed pro-apoptotic mechanisms of dexamethasone include direct activation of the pro-apoptotic members of the Bcl-2 family (e.g. Bcl-2, Bad and Bcl-XL [29]; suppression of cell survival factors such as AP-1 [30] or via interaction of the glucocorticoid receptor with the pro-apoptotic death-associated protein DAP3 [31]. Alternatively, indirect suppression of anti-apoptotic cytokines by corticosteroids may cause apoptosis in various cell types [26] and IL-6, a known proliferative stimulus for PASMC [32], may be important in our study. Dexamethasone-induced apoptosis may also vary depending on the stage of the cell cycle. For example in activated T cells, dexamethasone induced apoptosis in G0/G1 phase, but not in the proliferative phase [33]. It would be interesting to further examine the potential different effects through phases of the cell cycle in PASMC. Finally, dexamethasone may induce apoptosis via altered signalling of the key inflammatory transcription factor NF-κB [34].

The evidence that inflammation is associated with the pathogenesis of many types of PAH, including idiopathic PAH (iPAH) is increasing [35]. Histological specimens from patients demonstrate peri-vascular inflammatory infiltrates in subtypes of PAH including CHD [12], CTD-PAH, portopulmonary PH and more recently in iPAH patients [7]. These infiltrates involve T cells, monocytes/macrophages, DC, and, to a lesser extent, B cells [3, 7, 10–12]. Circulating levels of cytokine and chemokines are elevated in blood from iPAH patients, some of which predict worse outcomes [9].

Potential inflammatory pathways co-ordinating this inflammatory phenotype include the key transcription factor NF-κB. Indeed we demonstrated NF-κB activation in MCT-treated rat PASMC*.* NF-κB activation via IKK2 is implicated in a feed-forward inflammatory cascade involving the induction of key chemokines such as IL-8/CXCL-8 and cytokines including IL-1, IL-6 and TNF-α involved in the pathogenesis of iPAH [36, 37] [38]. Furthermore, recognised triggers of PAH including viral infections [39], oxidative stress [40] and alveolar hypoxia and shear stress [41, 42] are also activators of NF-κB [36]. Indeed, NF-κB activation was demonstrated in alveolar macrophages obtained at bronchoscopy in patients with less severe iPAH [43]; and in alveolar macrophages, lymphocytes, and pulmonary endothelial and PASMC in iPAH patients with severe end stage disease [5]. This would suggest a role for NF-κB throughout the disease process. Furthermore, constitutive activation of NF-κB has also been demonstrated in PASMC from patients with heritable PAH [44]. Finally, NF-κB activation has also been shown to be a feature of animal models including MCT-induced and sugen/hypoxia PH in rats [45–47]. Longitudinal studies in MCT-induced PH have documented NF-κB activation in vascular cells and mononuclear cells, with evidence for initial p65 nuclear binding followed by a more persistent reduction in the inhibitory subunit *IκB-α* from 2 weeks [45, 46].

Anti-inflammatory glucocorticoids have multiple mechanisms of action. To exert their anti-inflammatory effects, glucocorticoids bind the intracellular glucocorticoid receptor (GR), thereafter to specific DNA sequences (glucocorticoid-response elements, GRE) to induce transcription i.e. transactivation of anti-inflammatory genes, such as IκBα (the gatekeeper that limits NF-κB migration into the nucleus) [48]. A second and probably more important mechanism by which GC suppress inflammation is through direct GR protein-protein interaction thus inhibition of pro-inflammatory mediators, i.e. transrepression, as described between GR and p65 [49]. Transrepression explains the GC-mediated inhibition of many inflammatory cytokines (e.g. IL-6, IL-1β, TNF-α) which all have NF-κB and/or AP-1 elements in their gene promoter regions [50], and we demonstrated this as an example in PASMC with both TNF-induced IL-6 and CXCL8 release reduced following dexamethasone and NF-κB inhibition. In keeping with these effects, our results show a significant reduction in nuclear p65 as determined by TransAm assay in control human PASMC following dexamethasone treatment. However, the effect was small and does not account for the full effects of dexamethasone reported here and other down-stream actions of dexamethasone on NF-κB activation are also likely to be important. Transrepression of pro-inflammatory transcription factors is thought to largely explain the clinical success of GC as effective anti-inflammatory agents whereas transactivation is thought to contribute to the adverse metabolic effects of GCs [51].

There is a rationale for blocking NF-κB using dexamethasone or specific inhibitors of NF-κB. Indeed, in some patients with associated inflammatory conditions including SLE, MCTD and POEMS, treatment of the underlying condition with immunosuppression including GC improved pulmonary haemodynamics in PAH [14, 15, 52]; although this has been rarely studied in iPAH to date [18]. In vitro studies show that GC inhibit proliferation of human PASMC derived from patients with iPAH [20] in association with inhibition of G0/G1 to S phase cell cycle progression, inhibition of PDGF-induced PASMC migration [20] and reduced NF-κB activation [53]. Interestingly, other immunosuppressive agents including azathioprine, rapamycin and cyclosporin had no effect on PASMC proliferation [20]. We reproduced this anti-proliferative effect using dexamethasone in rat PASMC in association with a reduction in IL-6 and an increase in BMPRII mRNA [19].

NF-κB inhibition has been shown to exert both anti-apoptotic and pro-apoptotic effects in different cell types [34] and we show in this study that an IKK-2 inhibitor increased apoptosis in human PASMC *in vitro*. NF-κB blocks cell death through induction of anti-apoptotic and antioxidant genes and is an essential ‘survival factor’ for immune cells. Anti-apoptotic NF-κB target genes include caspase-8-c-FLIP, cellular inhibitors of apoptosis (c-IAPS), A1 (also known as Bfl1), TNFR-associated factor 1 (TRAF1), TRAF2. Caspase-8-c-FLIP, cIAPS and A1, which all act in concert to block multiple steps in apoptotic signaling [34]. In contrast, NF-κB suppresses apoptosis in Ras-transformed cells and other tumours. This is considered a hallmark of cancer and pro-survival roles for various IKK family members have been reported [54]. Inhibition of NF-κB using the proteasome inhibitor bortezomib enhanced apoptosis and tumour regression in response to chemotherapy [55]. Ultimately, the pro- or anti-apoptotic effects of NF-κB, as with corticosteroids, are cell and stimulus-specific [54]. That NF-κB can suppress apoptosis in cancer cell lines is being explored as a tool in cancer therapies, and could be of potential relevance to PH where excessive cell proliferation and reduction in apoptosis has been likened to a cancer like phenotype [56].

In models of PH, both GC and specific inhibitors of NF-κB prevent the onset of PH [45, 57–60] and reverse established PH, for example in the MCT model [19, 46]. The reverse-remodelling hypothesis then fits with our finding that both dexamethasone and IKK2 inhibition increased apoptosis in relevant cells suggesting that inhibition of NF-κB led to a regression of the pulmonary smooth muscle layer in the *in vivo* studies. NF-κB indeed regulates aspects of control of cell turnover including apoptosis [61, 62]. Of course to assess a causal link between inhibition of NF-κB and an increase in PASMC apoptosis would require further in-depth studies using transgenic mice and knockout models. Induction of apoptosis as a reverse-remodelling approach has been shown in previous PAH studies investigating nitric oxide [63], dichloroacetate [64], imatinib [65] and statins [66]. That inhibition of NF-κB could be augmenting apoptosis in PASMC is relevant to PAH therapies. In the rat sugen-hypoxia PAH model, administration of the NF-κB inhibitor pyrrolidine dithiocarbamate (PDTC) also reduced apoptosis of PASMC and obliteration of these vessels, promoted immune regulation (e.g. by increasing perivascular T cells and reducing T lymphocytes and CD45RA+ B lymphocytes, while preserving right ventricular function [47]).

In addition to the pro-apoptotic effect of NF-κB inhibition via direct effects on apoptotic pathways as mentioned above, suppression of inflammatory mediator release may also modulate vascular remodelling. IL-6 is implicated in human PAH [8, 9] and rodent PH models [67] [68] and has been shown to be pro-proliferative in vascular SMC [32]. Hagen et al. identified a regulatory loop involving IL-6 and BMP signalling, in which upregulated IL-6 was seen in mice PASMC lacking functional BMPR-II, and IL-6 administration *in vivo* increased BMP pathway activity [69]. Inhibiting IL-6 expression could, therefore, augment the anti-proliferative ‘damping’ effects of BMP signalling. Furthermore, pulmonary arterial EC co-treated with IL-6 and MCT *in vitro* increased proliferation due to enhanced activation of STAT3 [70]. Persistent activation of STAT3 has been shown to reduce BMPR-II expression [71]. We provide further evidence of such a regulatory loop here as we show that STAT3 activation, as measured by phosphorylation and by absolute levels, increased with MCT treatment but was reversed by dexamethasone and have previously reported that MCT and dexamethasone inversely modulate BMPR2 mRNA expression in this model [19].

In a gastric cancer cell line, IL-6 inhibited apoptosis through inhibition of JNK signalling and subsequent cell death [72]. It would therefore be of interest to ascertain whether, in addition to reducing proliferation, whether blocking IL-6 could directly augment apoptosis in PASMC. It is interesting that a recent study by Farkas et al., inhibiting IL-6 with an anti-IL-6 antibody had a lesser effect on PASMC apoptosis than using PDTC to inhibit NF-κB [47], suggesting this approach is likely to be more beneficial than focusing on a single pathway.

### Limitations

We were limited by not performing all analyses on human PASMC, and it would be ideal to do this on PASMC isolated from patients. This is however difficult as the only available lung samples are from patients with end-stage pulmonary hypertension following transplantation or death. We therefore extrapolated findings from PASMC isolated from healthy lung. We did not include a dexamethasone alone arm within the in vivo study, and so the effects of dexamethasone on baseline hemodynamics and vascular remodeling is unknown.

### Summary

We showed by several methods that in rat and human PASMC, dexamethasone and specific inhibition of NF-κB increased PASMC apoptosis, and reduced IL-6 and CXCL8 release from these cells. In human PASMC, dexamethasone reduced p65 nuclear import and/or DNA binding as measured by TransAm assay. These results suggest that dexamethasone exerted anti-remodeling effects on PASMC in the rat MCT PH model through an induction of apoptosis in these cells, which is likely to be through NF-κB inhibition. Which NF-κB signalling pathway components, and whether related to a reduction in IL-6 and CXCL8 signaling, requires further study.

### Clinical implications

The anti-remodelling effects of dexamethasone in the MCT model provide a rationale for clinical trials using GC and/or NF-κB inhibitors in patients with PAH including iPAH. That IKK-2 inhibition exerted similar effects as dexamethasone on cytokine release and pro-apoptotic effects, at least in control human cells, is encouraging as these agents are likely to have less systemic side effects than GC, although they may potentially lead to opportunistic infections due to suppression of the innate immune system with chronic treatment.
